# Liquid–Liquid Criticality in TIP4P/2005 and Three-State Models of Water

**DOI:** 10.1021/acs.jpcb.3c00696

**Published:** 2023-04-25

**Authors:** Claudio A. Cerdeiriña, Diego González-Salgado, Jacobo Troncoso

**Affiliations:** Instituto de Física e Ciencias Aeroespaciais da Universidade de Vigo and Unidad MSMN Asociada al CSIC por el IQFR, Ourense 32004, Spain

## Abstract

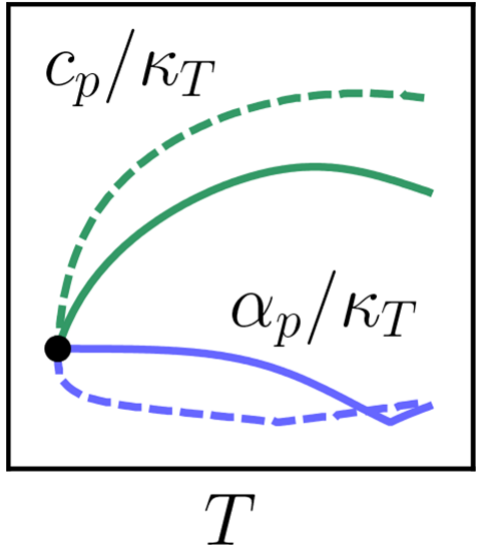

Molecular dynamics simulations leading to the isothermal compressibility, the isobaric thermal expansivity, and the isobaric heat capacity of TIP4P/2005 water are found to be consistent with the coordinates of its second, liquid–liquid critical point reported recently by Debenedetti et al. [Science2020, 369, 289−29232675369]. In accord with the theory of critical phenomena, we encounter that the rise in the magnitude of these response functions as temperature is lowered is especially marked along the critical isochore. Furthermore, response-function ratios provide a test for thermodynamic consistency at the critical point and manifest nonuniversal features sharply distinguishing liquid–liquid from standard gas–liquid criticality. The whole pattern of behavior revealed by simulations is qualitatively the same as the one of a three-state Ising model of water exhibiting a low-temperature liquid–liquid critical point. Exact solutions for the two-state components of such a three-state model are also provided.

## 1 Introduction

The existence of a liquid–liquid transition for supercooled water has stirred a large amount of research since it was firmly hypothesized.^1^ The transition would end at an upper critical point located at a temperature lower than the ice homogeneous nucleation temperature, namely, ∼232 K at atmospheric pressure. Since below this temperature rapid crystallization prevents probing the supercooled liquid phase in typical experiments, most evidence restricts to the one-phase sharp rises in the magnitude of response functions when temperature is lowered—as earlier uncovered^2−4^ and supplemented by the latest work reporting maxima consistent with a liquid–liquid critical point.^5−7^

Part of the difficulties are overcome by molecular simulation since for a number of force fields it offers characteristic time scales for crystal nucleation and relaxation enabling the liquid phase at deeply supercooling conditions to be probed.^8^ Accordingly, liquid–liquid criticality has been reported for the ST2, E3B3, iAMOEBA, TIP4P/2005, TIP4P/Ice, and various ab initio models of water.^9−16^ Attention has been mostly focused, like in experiments, on the one-phase region.

The one-phase region is indeed the target of Debenedetti et al. work^14^ characterizing the liquid–liquid critical point of the two TIP4P variants mentioned above. Their study allowed a concrete estimation of critical coordinates and, furthermore, yielded values for critical exponents in accord with the accepted ones for the universality class of the three-dimensional Ising model (note also ref (17)). This is the starting point of the present work, in which we aim to thoroughly analyze the behavior of the isothermal compressibility κ_*T*_, the isobaric thermal expansivity α_*p*_, and the isobaric heat capacity per particle *c*_*p*_ as the critical state specified by Debenedetti et al. is approached from the one-phase region.

To do so, we perform molecular dynamics simulations for TIP4P/2005 water at conditions of temperature *T* and pressure *p* allowing us to analyze κ_*T*_(*T*), α_*p*_(*T*), and *c*_*p*_(*T*) along the critical isobar, *p* = *p*_*c*_, and the critical isochore, ρ = ρ_*c*_. Such simulations indicate that critical anomalies are, as earlier pointed out on theoretical grounds,^18^ larger for the critical isochore. They also suggest a thermodynamic consistency test at criticality based on the various ratios between κ_*T*_, α_*p*_, and *c*_*p*_.

Our simulations do not allow, however, approaching the liquid–liquid critical point asymptotically. To fill such a crucial gap, we use a three-state Ising model—henceforth to be referred to as the “TS model”—describing the thermodynamic scenario in which water possesses two critical points, gas–liquid and liquid–liquid.^19^ The model yields full qualitative consistency with TIP4P/2005 water even at the mean-field level.

Further characterization of TIP4P/2005 liquid–liquid criticality entails comparison against gas–liquid criticality, which we undertake with the aid of literature experimental data.^20^ In addition, we consider separately the exact solutions of the two two-state components of our TS model, namely, a standard lattice gas SLG^21^ and a compressible cell CC model.^22^ This makes sense taking into account, on the one hand, the wide recognition of the SLG as a suitable model for gas–liquid criticality and, on the other, that the CC model agrees with phenomenological approaches to scaling in one-component liquid–liquid criticality.^23,24^

The paper is organized as follows. In section 2 we briefly describe our simulation methods as well as the TS model. Results are presented and discussed in section 3. In subsections 3.1 to 3.3 we appeal to the mean-field solutions of the three-state model, while subsection 3.4 focuses on the exact solutions of its individual SLG and CC components and is supplemented by an Appendix. Section 4 contains a few concluding remarks.

## 2 Methods

### 2.1 TIP4P/2005 Water

TIP4P/2005 is an all-atom rigid model of water with Lennard-Jones and electrostatic interactions. Details characterizing the site geometry and its associated energetic parameters were reported originally.^25^ The model has proved quite accurate and, as such, it is widely used for a variety of applications.

Standard *NpT* molecular dynamics simulations^26,27^ for *N* = 500 TIP4P/2005 molecules were conducted using GROMACS^28^ (version 2022.1). A cubic box under periodic boundary conditions was used. Pressure and temperature were controlled using a Parrinello–Rahman barostat^29^ and a Nosé–Hoover thermostat^30,31^ with time constants set to 31.14 and 5.17 ps, respectively.^14^ Including usual long-range corrections, Lennard-Jones interactions were truncated at a 1 nm cutoff radius. Coulombic interactions were computed using the Particle Mesh Ewald summation scheme^32^ with the same cutoff radius for the Lennard-Jones interactions and the real part of Ewald sums. A fourth degree interpolation with a 0.1 nm grid was used for the reciprocal space sum. The time constant used for the integration of equations of motion was 0.002 ps and the overall simulation time 2 μs.

Our raw data are the volume per particle *v* and enthalpy per particle *h* for each thermodynamic state, characterized by a prescribed pair of *T* and *p* values. Then, starting from the definitions
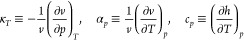
1an incremental procedure for evaluating the corresponding derivatives over *T* and *p* intervals ranging from 5 to 10 K and from 100 to 200 bar is used to get κ_*T*_(*T*, *p*), α_*p*_(*T*, *p*), and *c*_*p*_(*T*, *p*). These yield via exact thermodynamic relations the isentropic compressibility and the isochoric heat capacity per particle
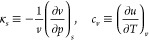
2where *u* stands for the internal energy per particle.

Conditions of *T* and *p* for simulated states were chosen on the basis of the TIP4P/2005 critical coordinates reported by Debenedetti et al.,^14^ namely, *T*_*c*_ ≃ 171.5 K, *p*_*c*_ ≃ 1872 bar, and ρ_*c*_ ≃ 1.024 g cm^–3^. Thus, simulations from 185 to 300 K at 1772, 1872, and 1972 bar yielded κ_*T*_(*T*), α_*p*_(*T*), and *c*_*p*_(*T*) along the critical isobar. Note that the incremental calculation of derivatives in eq 1 shortens the *T* or *p* intervals at which κ_*T*_, α_*p*_, and *c*_*p*_ can be evaluated, implying for instance that no reliable α_*p*_ and *c*_*p*_ data below 190 K could be obtained. On the other hand, determination of κ_*T*_(*T*), α_*p*_(*T*), and *c*_*p*_(*T*) data along the critical isochore was made from simulations along isobars within the 350 to 1500 bar interval at the relevant temperatures for each isobar allowing to meet the ρ = ρ_*c*_ condition. More specifically, a preliminary calculation led to the interpolated temperature value corresponding to ρ = ρ_*c*_, , so that the corresponding incremental procedure yielded κ_*T*_, α_*p*_, *c*_*p*_, and derived properties for the corresponding  state. In general, no reliable response-function data below 190 K could be obtained. The primary *v*(*T*, *p*) and *h*(*T*, *p*) data are deposited in tabulated form as Supporting Information.

It is important to note that Debenedetti et al. were able to prove TIP4P/2005 liquid–liquid criticality by performing simulations down to 177 K. This required simulations of up to 100 μs in boxes containing up to 36 424 molecules. In this context, they claimed that getting closer to criticality (e.g., down to 173 K) is not feasible even for the long simulation times and large box sizes they used. Our approach, recall, simulations of 2 μs for a box containing 500 molecules, enables reliable *v* and *h* data down to 190 K to be obtained while such a low temperature could only be extended down to 185 K by averaging out over five independent simulations to get an overall simulation time of 10 μs. As advanced in the Introduction, these limitations prevent reaching the full asymptotic critical behavior and certainly motivate appealing to the three-state model to be described next.

### 2.2 Three-State Ising Model

As noted in the Introduction, this model combines the two-state SLG^21^ with a two-state compressible cell CC model.^22^ Thus, with three basic states for cells accommodating these two-state components, the resulting model—which pertains to the “Blume-Capel” or “Blume–Emery–Griffiths” class explored since long ago^33−35^ and currently adopted to explain water’s unusual thermodynamics^19,36,37^—characterizes local energetic, volumetric, and entropic effects with the aid of two energetic parameters, ε_0_ and *δε*, two volumetric parameters, *v*_0_ and *δv*, and one entropic parameter λ. Here we employ the parameter values proposed originally, which at the mean-field level lead to an upper liquid–liquid critical point and a standard gas–liquid one with coordinates *T*_*c*_ ≃ 162.37 K, *p*_*c*_ ≃ 1700 bar, and ρ_*c*_ ≃ 0.903 g cm^–3^ and *T*_*c*_ ≃ 623.74 K, *p*_*c*_ ≃ 60 bar, and ρ_*c*_ ≃ 0.512 g cm^–3^, respectively.^19^

**Figure 1:**
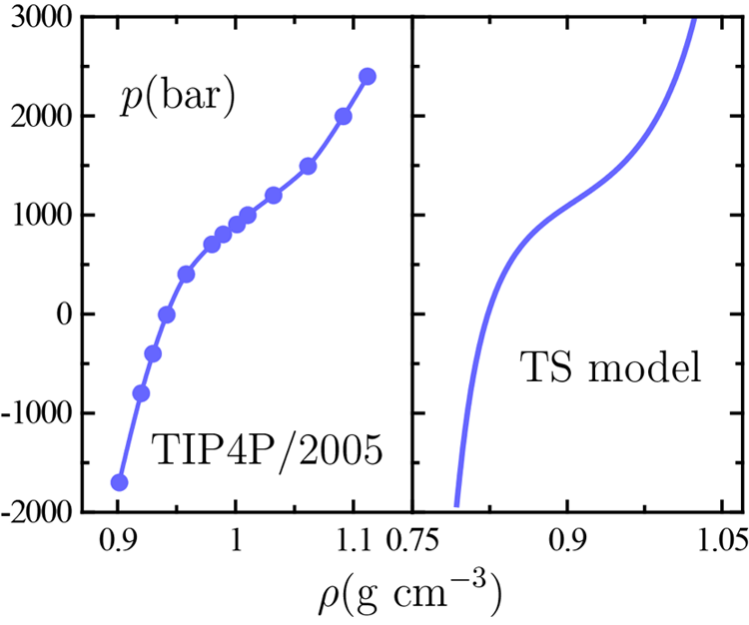
TIP4P/2005 isotherm in the pressure–density *p*–ρ plane. The left panel shows literature simulation data at the temperature *T* ≃ 207 K,^38^ the right one, three-state (TS) model values at *T* = 200 K.

The model has been shown to reproduce water’s “two-critical-point scenario” in detail.^19^ As a further illustration of its capabilities, Figure 1 shows that the shape of a model’s low-temperature—while supercritical—*p*(ρ) isotherm exhibits a close resemblance to TIP4P/2005 simulation data. It is to be noted that the TIP4P/2005 curve in Figure 1 is in dramatic contradiction with the class of van der Waals equations of state, for which a supercritical isotherm is strictly incompatible with states of negative pressure. The nice accord in Figure 1 then constitutes an indication that the augmented equation of state provided by our three-state picture meets challenges demanded by supercooled water.

## 3 Results and Discussion

### 3.1 Critical Isochore

**Figure 2:**
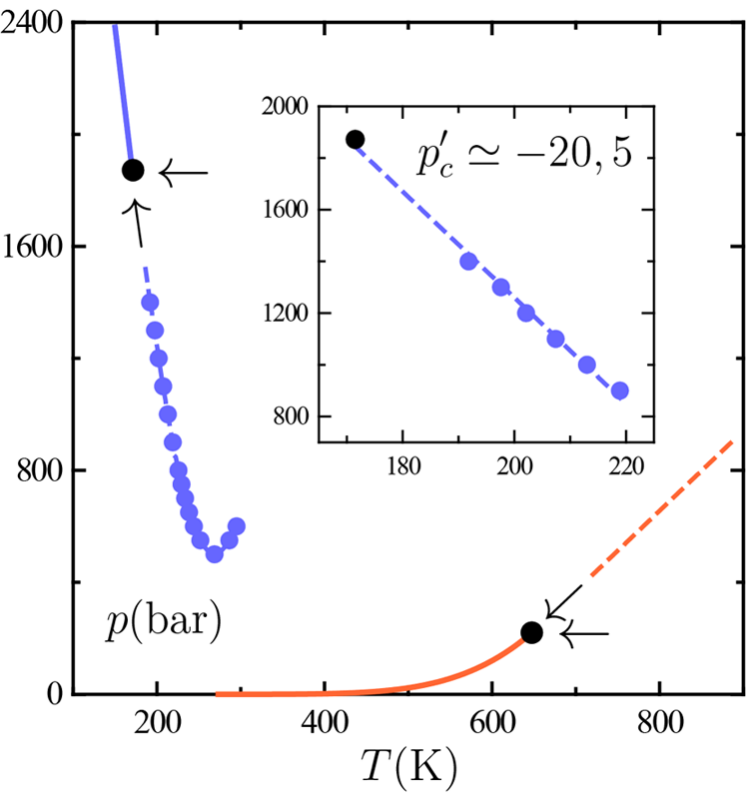
Fluid phase behavior of water in the pressure–temperature *p*–*T* plane. Dashed curves and arrows serve to indicate the critical isochore and critical isobar on approaching the TIP4P/2005 liquid–liquid critical point (black circle at low *T*) and the experimental gas–liquid one (black circle at high *T*). Gas–liquid experimental data (orange lines) are taken from the literature.^20^ TIP4P/2005 liquid–liquid behavior is based on our simulation data (blue circles), with the liquid–liquid coexistence line (solid line) being extrapolated as the limiting slope of the critical isochore *p*_*c*_^′^.^39^ The inset is an enlarged plot of TIP4P/2005 data close to liquid–liquid criticality yielding *p*_*c*_^′^ ≃ −20.5 bar K^–1^, while for gas–liquid criticality *p*_*c*_^′^ ≃ 2.8 bar K^–1^.^40^

Figure 2 illustrates the two paths of approach to the critical point in the *p*–*T* plane under question, recall, the critical isochore and the critical isobar. The plotted data yield an estimation of the limiting slope of the critical isochore at criticality *p*_*c*_^′^, which shall further appear throughout our discussion as a relevant parameter.

**Figure 3:**
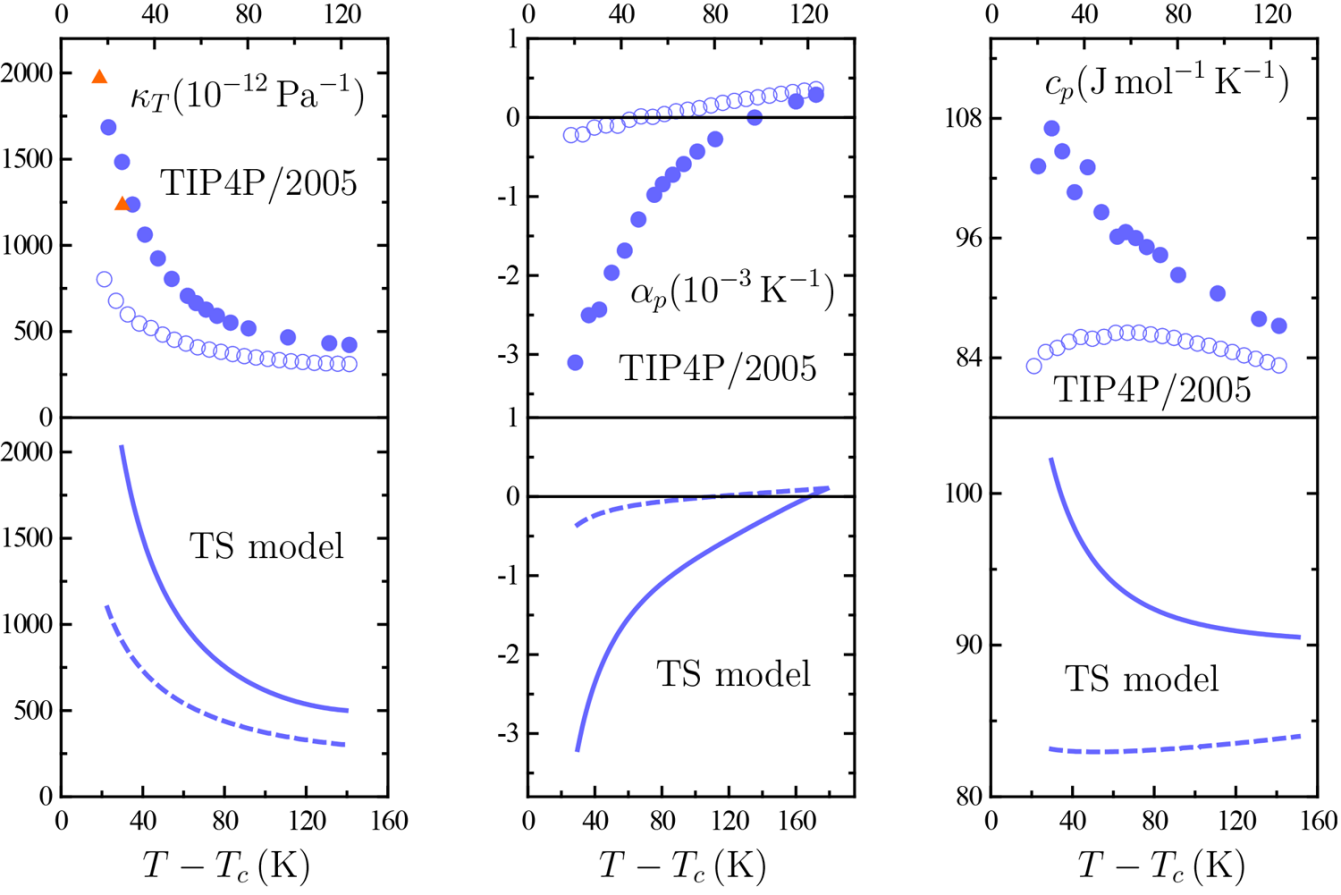
Isothermal compressibility κ_*T*_, isobaric thermal expansivity α_*p*_, and isobaric heat capacity per particle *c*_*p*_ on approaching the liquid–liquid critical point occurring at the temperature *T*_*c*_. Filled circles are our TIP4P/2005 simulated values along the critical isochore, open ones are their counterparts along the critical isobar, and triangles are Debenedetti et al. data at ρ ≃ 1.012 g cm^–3^.^14^ Continuous lines in lower panels are our three-state (TS) model values along the critical isochore, dashed ones being their counterparts along the critical isobar. The *c*_*p*_ values have been deliberately shifted by a constant 70 J mol^–1^ K^–1^ value (see text).

Figure 3 shows TIP4P/2005 κ_*T*_, α_*p*_, and *c*_*p*_ data along the two paths. Comparison with κ_*T*_ data along a near-critical isochore from ref (14)—obtained as the *q* = 0 limit of the structure factor—reveals mutual consistence. While ref (14) provides data down to 177 K enabling κ_*T*_ critical behavior to be probed, it is to be recalled here that the details of our simulations specified in subsection 2.1 rule out the possibility of obtaining reliable data below 190 K. Although this limitation precludes an asymptotic approach to *T*_*c*_, the behavior of the three response functions under question is consistent with diverging behavior at criticality. And the relevant point in this connection is that the rise in response functions as temperature is lowered down to *T*_*c*_ is steeper along the critical isochore. Such a “critical-isochore-induced enhancement” was predicted long ago and is certainly contemplated by standard scaling theory.^18^

This may be expressed in terms of critical exponents embodied in the power law
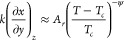
3where *x* can be *v* or *s*, *k* can be ±1/*v*_*c*_ or *T*_*c*_, and *y* and *z* can be *T* or *p*, while ψ stands for the corresponding critical exponent and *A*_*r*_ for the corresponding critical amplitude (with *r* correspondingly being α_*p*_, κ_*T*_, or *c*_*p*_). It is well-known that ψ = γ ≃ 1.239 along the critical isochore, while along the critical isobar ψ = 1 – β/Δ ≃ 0.792 < γ, with β ≃ 0.326 and Δ ≃ 1.556. Since divergences are “stronger” for larger ψ, it is a result of the theory of critical phenomena that the critical enhancement of α_*p*_, κ_*T*_, or *c*_*p*_ is more marked along the critical isochore. It is to be emphasized that consistent with this are TIP4P/2005 simulation data in Figure 3, which also shows that our three-state (TS) model exhibits the same picture.

### 3.2 Thermodynamic Consistency

**Figure 4:**
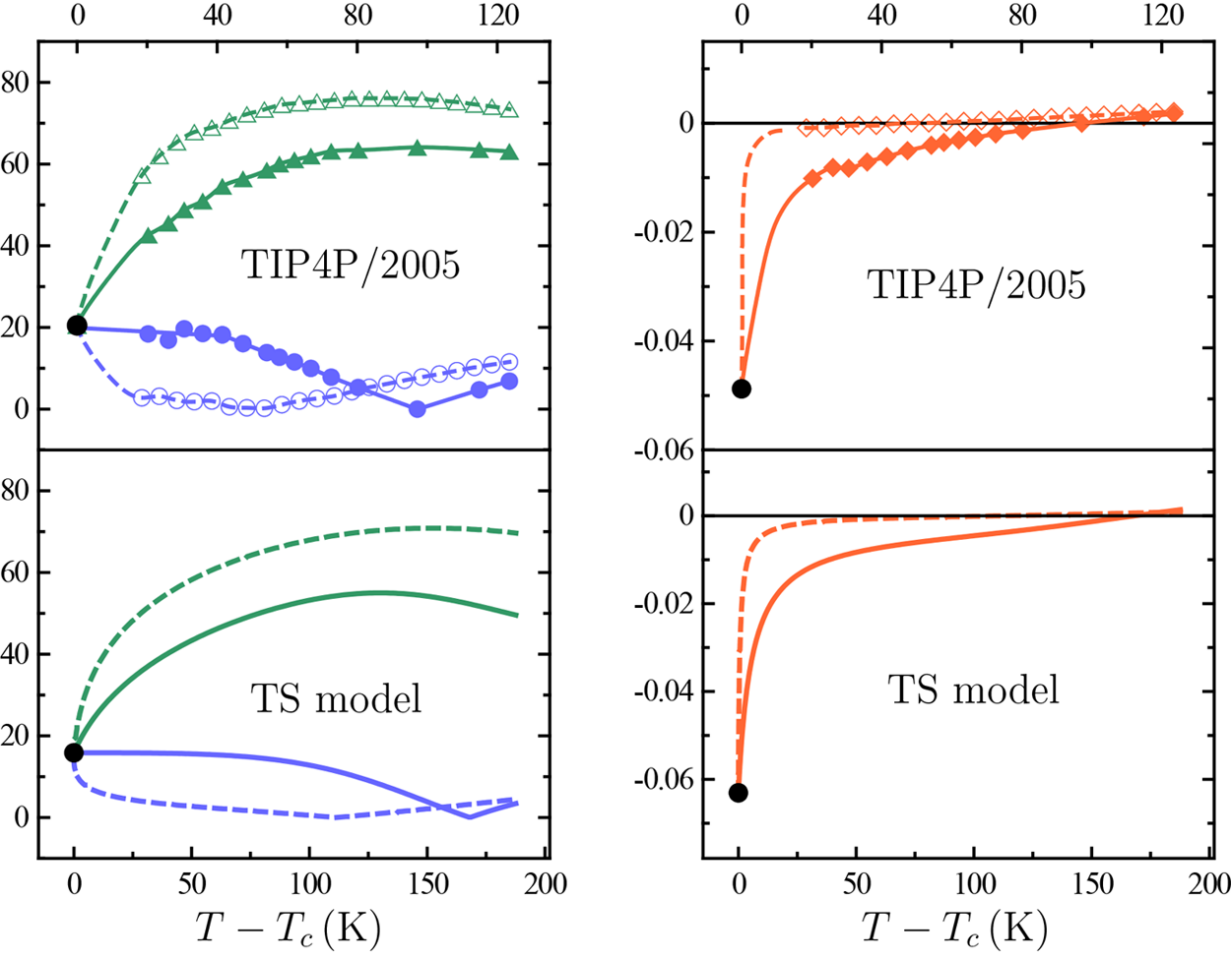
Response-function ratios |(*∂p*/*∂T*)_*v*_| (circles, purple),  (triangles, green), and (*∂T*/*∂p*)_*s*_ (diamonds, orange) on approaching the liquid–liquid critical point occurring at the temperature *T*_*c*_. The black filled circles are the corresponding critical values, identified to |*p*_*c*_^′^| in left panels and to  in the right ones (cf. Figure 2). Filled symbols are our TIP4P/2005 simulated values along the critical isochore, open ones being their counterparts along the critical isobar. Lines between our simulation points and the critical one are interpolated values. Continuous lines in lower panels are our three-state (TS) model values along the critical isochore, dashed ones being their counterparts along the critical isobar.

Figure 4 shows the temperature dependence of |(*∂p*/*∂T*)_*v*_|, , and (*∂T*/*∂p*)_*s*_ as obtained from our TIP4P/2005 α_*p*_, κ_*T*_, and *c*_*p*_ simulated data via the following exact thermodynamic relations
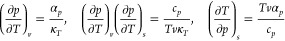
4Since κ_*T*_, α_*p*_, and *c*_*p*_ all diverge with the same critical exponent, it comes from the combination of eqs 3 and 4 that |(*∂p*/*∂T*)_*v*_|, , and (*∂T*/*∂p*)_*s*_ reduce in the asymptotic region to ratios between critical amplitudes that, as such, take on finite values at the critical point. This allows the following test for thermodynamic consistency at criticality.

Consider the exact thermodynamic relation
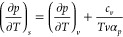
5and note that *c*_*v*_ is a “weakly diverging” response function that as such has a small critical exponent ψ = α = 0.109 while, recall, ψ = γ = 1.239 for the “strongly diverging” α_*p*_. Accordingly, the *c*_*v*_/*Tvα*_*p*_ term in the right-hand side of eq 5 becomes negligible relative to (*∂p*/*∂T*)_*v*_ when *T* → *T*_*c*_. It then turns out that the critical state demands (*∂p*/*∂T*)_*s*_ = (*∂p*/*∂T*)_*v*_. Figure 4 shows that our simulation data are not inconsistent with this: with the |(*∂p*/*∂T*)_*v*_| value at criticality identified to |*p*_*c*_^′^| as obtained from the critical isochore in Figure 2, one infers that it is likely that  also tends to |*p*_*c*_^′^| as *T* → *T*_*c*_—and, hence, (*∂p*/*∂T*)_*s*_ = (*∂p*/*∂T*)_*v*_.

Figure 4 shows that fulfillment of this critical-point-related constraint requires the expediency of getting closer to *T*_*c*_ than our simulations permit. Nevertheless, also evident from Figure 4 is the suggestion that our TS model settles the issue, thereby providing a guide as to what should be expected for TIP4P/2005 water. Further simulations allowing us to estimate the corresponding ratios closer to *T*_*c*_ seem worthwhile. They would be especially suited for (*∂T*/*∂p*)_*s*_, for which a closer approach to criticality is expected to reveal a dramatic temperature dependence.

Note finally that the TS model picture for  and (*∂T*/*∂p*)_*s*_ in Figure 4 is found as soon as *c*_*p*_ data are shifted by a constant 70 J mol^–1^ K^–1^ value (cf. Figure 3). This partially corrects the unrealistically underestimated values originating from the coarse-grained nature of the model, which precludes any detailed approach to the variety of degrees of freedom contributing to *c*_*p*_ at low temperatures.

### 3.3 Nonuniversal Features

**Figure 5:**
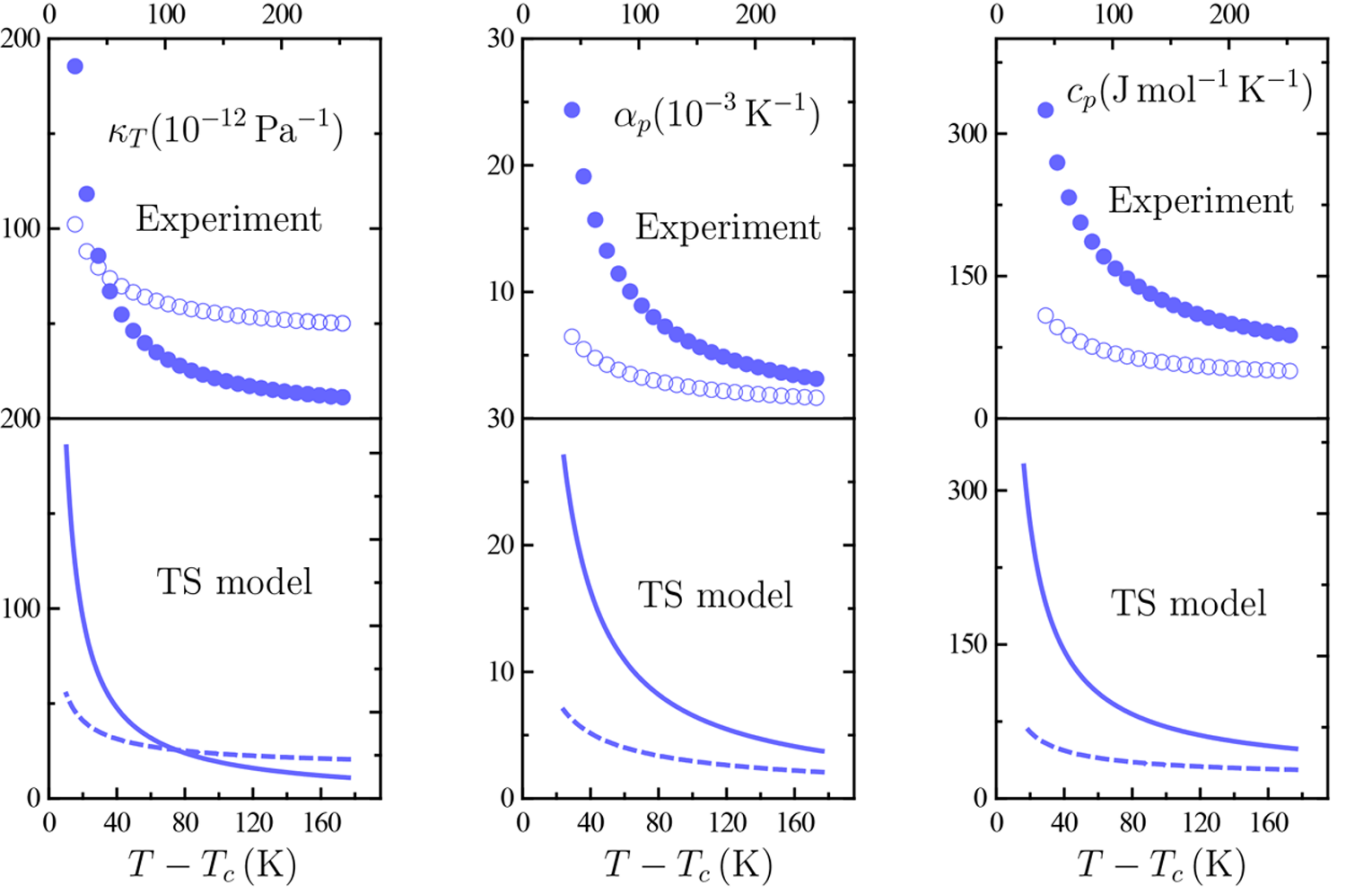
Isothermal compressibility κ_*T*_, isobaric thermal expansivity α_*p*_, and isobaric heat capacity per particle *c*_*p*_ on approaching the gas–liquid critical point occurring at the temperature *T*_*c*_. Filled symbols are water’s literature experimental data along the critical isochore, open ones being their counterparts along the critical isobar.^20^ Continuous lines in lower panels are our three-state (TS) model values along the critical isochore, dashed ones being their counterparts along the critical isobar.

Further characterization of water’s liquid–liquid criticality involves comparison with gas–liquid criticality, a task we undertake by incorporating in our analysis literature experimental data near water’s gas–liquid critical point—and, for completeness, results from our TS model. Figure 5 shows κ_*T*_(*T*), α_*p*_(*T*), and *c*_*p*_(*T*) data along the gas–liquid critical isobar and critical isochore (cf. Figure 2). Comparison with Figure 3 reveals a quite similar picture, with the main differences being the opposed sign of α_*p*_ and the crossing of κ_*T*_ curves corresponding to the critical isochore and the critical isobar for gas–liquid criticality. One concrete manifestation of the similar patterns of behavior observed are the common values of critical exponents in eq 3 and the underlying common universality class both transitions belong to. By contrast, significant differences at a quantitative level are evident, implying significantly larger critical amplitudes in eq 3 for gas–liquid criticality.

**Figure 6:**
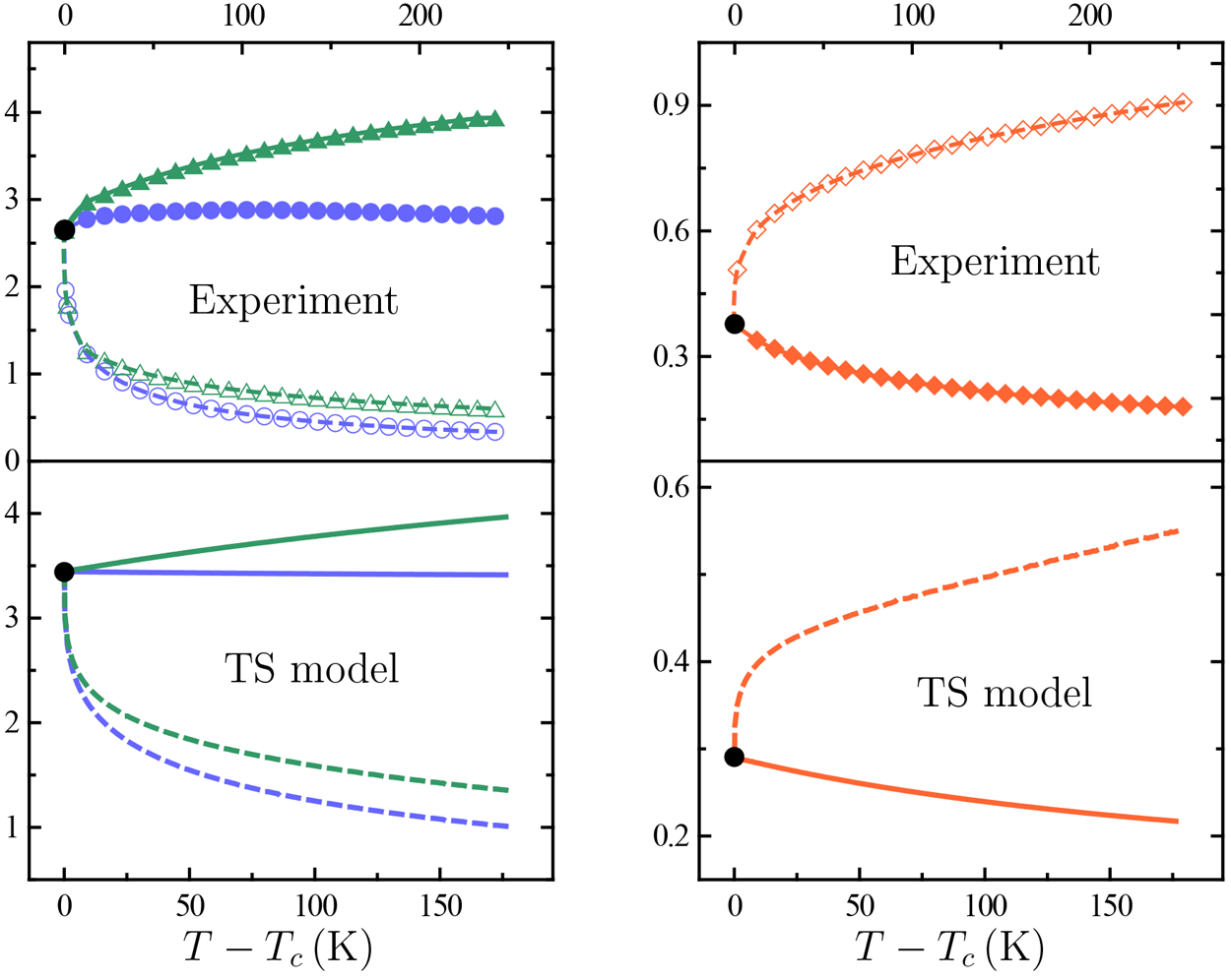
Response-function ratios (*∂p*/*∂T*)_*v*_ (circles, purple),  (triangles, green), and (*∂T*/*∂p*)_*s*_ (diamonds, red) on approaching the gas–liquid critical point occurring at the temperature *T*_*c*_. The black circles are the critical values, identified to *p*_*c*_^′^ in left panels and to  in the right ones (cf. Figure 2) Filled symbols are water’s literature experimental data along the critical isochore, open ones being their counterparts along the critical isobar.^20^ Continuous lines in lower panels are our three-state (TS) model values along the critical isochore, dashed ones being their counterparts along the critical isobar.

Comparison of gas–liquid (*∂p*/*∂T*)_*v*_, , and (*∂T*/*∂p*)_*s*_ data in Figure 6 with their liquid–liquid counterparts in Figure 4 evidences sharply contrasting pictures on both quantitative and qualitative grounds. Recall that these response-function ratios are in effect ratios between critical amplitudes in eq 3, implying that the nonuniversal (i.e., system-dependent) character of critical amplitudes may distinguish gas–liquid from liquid–liquid criticality.

**Figure 7:**
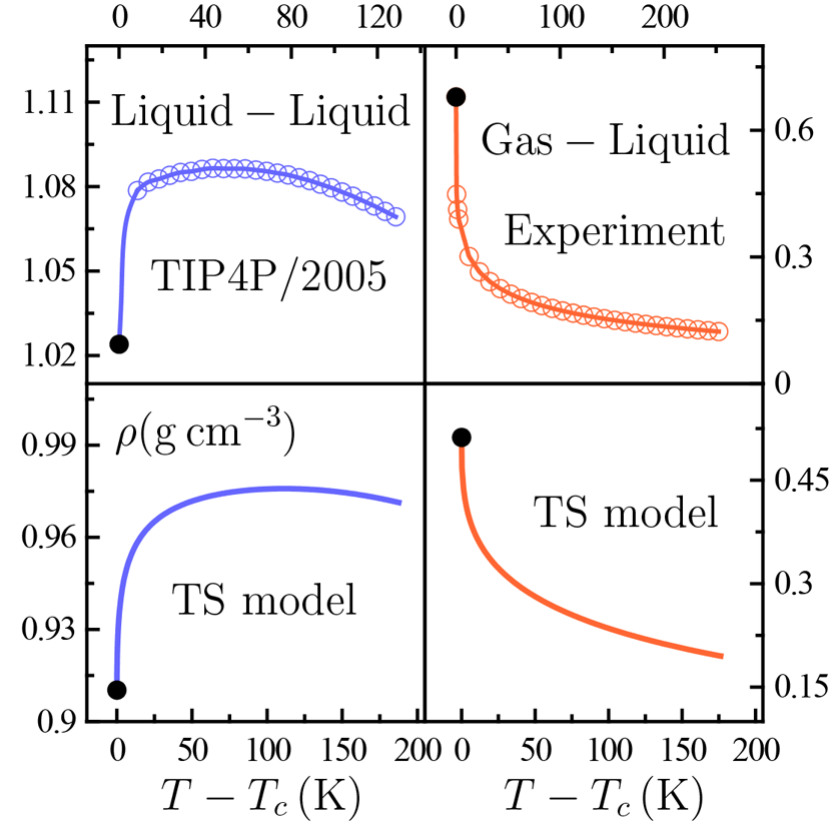
Temperature dependence of the density ρ along the critical isobar on approaching the TIP4P/2005 liquid–liquid and gas–liquid critical points occurring at *T*_*c*_. The black point in each panel is the corresponding critical value taken from the literature.^14,20^ Liquid–liquid TIP4P/2005 data above *T*_*c*_ are taken from the present simulations, the experimental water’s gas–liquid ones being taken from the literature.^20^ Lines between our simulation points and the critical one are interpolated values. Corresponding behavior for our three-state (TS) model is also illustrated.

To understand (*∂p*/*∂T*)_*v*_ behavior, it is useful to look at the functional form the mean-field *pρT* equation of state of the CC and SLG components our TS model reduces to. Since both have the mathematical structure *p* = *k*_*B*_*Tf*(ρ) + *g*(ρ), with *f*(ρ) a monotonically increasing function,^22,41^ it turns out that (*∂p*/*∂T*)_*v*_ reflects the variation of ρ at near-criticality. The joint inspection of data in Figures 4 to 6 and ρ(*T*) data in Figure 7 supports this.

When it comes to (*∂T*/*∂p*)_*s*_, we put the focus on eq 5. A remarkable difference between the liquid–liquid and gas–liquid cases is that the significantly smaller |α_*p*_| values for the former result in substantially larger *c*_*v*_/*Tvα*_*p*_ values. Such enhanced liquid–liquid *c*_*v*_/*Tvα*_*p*_ values largely dominate over the (*∂p*/*∂T*)_*v*_ contribution, while its temperature dependence is determined by the one of α_*p*_ [we have checked that the *c*_*v*_(*T*) dependence is insignificant not very close to criticality]. The net result is that, as Figure 4 evidences, (*∂T*/*∂p*)_*s*_ reflects α_*p*_ behavior. This is not the case for gas–liquid criticality, in which (*∂p*/*∂T*)_*v*_ and (*∂T*/*∂p*)_*s*_ take on values of the same order of magnitude owing to the larger |α_*p*_| values.

### 3.4 Asymptotic Behavior

An enhanced approach to asymptotic critical behavior demands exact solutions beyond mean-field approximation. This can be explored for CC and SLG models via their individual mappings into the exactly soluble spin-1/2 (two-state) Ising model. This is described in the Appendix, which contains full details on definitions and algebraic calculations.

The procedure explained in connection with eq A.14, leads to the critical amplitudes of response functions along the CC critical isochore (cf. eq 3):
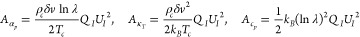
6where *k*_*B*_ stands for the Boltzmann constant while *Q*_*I*_ and *U*_*I*_ are critical amplitudes of the underlying Ising model. Equation 4 then readily yields
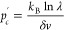
7Corresponding results for the SLG follow from eq A.19:
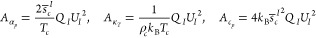
8
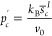
9where  is the critical entropy per spin of the underlying Ising lattice.

A few comments regarding signs and magnitudes are in order. While eq 9 prescribes *p*_*c*_^′^ > 0 for gas–liquid criticality as universally observed, the possibility that λ is greater or lesser than 1 results from eq 7 in *p*_*c*_^′^ unrestricted in sign for liquid–liquid criticality. Simulations of the Jagla and related core-softened model fluids support such unrestricted *p*_*c*_^′^,^24,42^ whereas the *p*_*c*_^′^ < 0 value of TIP4P/2005 reported in this work as well as the one of ST2 water^43^ demand λ < 1 (so that  from eq 6). As regards magnitudes, it comes out from eqs 6 and 8 that . Such an enhanced  value for the gas–liquid case is consistent with evidence from experiment and molecular simulation noted in subsection 3.3.

## 4 Concluding Remarks

Recent work by Jedrecy et al.^44^ has surprisingly reported the lack of coexistence of two liquid phases below Debenedetti et al.’s *T*_*c*_ reported value. In this connection, we emphasize from our present Figure 4 the close resemblance between TIP4P/2005 and a TS model exhibiting a liquid–liquid critical point terminating a coexistence line.^19^ Since it seems difficult to conclude that such an agreement is merely accidental, it is natural to expect two-liquid coexistence for TIP4P/2005 water. This issue needs to be clarified.

When it comes to the real existence of a liquid–liquid critical point for water, one is naturally led to the recent state-of-the-art spectroscopic study reporting the observation of two coexisting liquid phases between 195 and 215 K.^45^ Nevertheless, a detailed experimental analysis of thermodynamic or transport properties in the critical region is still not feasible for the short observation time imposed by crystal nucleation.^46^ A most promising approach continues to be the original one of analyzing the behavior in the one-phase region at temperatures as low as possible.^2−4^ While progress along this line has been made over the last years,^5−7^ the results of subsection 3.1 suggest that the experimental search of a supercooled-water isochore exhibiting the known attributes of a critical isochore is worthwhile.

## Appendix: Asymptotic Behavior of CC and SLG Models

We start from the two-state (spin-1/2) Ising model. Let, *K* ≡ *J*/*k*_*B*_*T*, and *h* ≡ *H*/*k*_*B*_*T* denote dimensionless thermodynamic variables of a system of  spins occupying the sites of a regular lattice at a temperature *T* in the presence of an external magnetic field *H*; *F* is the free energy, and *J* is the spin coupling energy. On introducing the dimensionless energy per spin  and magnetization per spin , thermodynamics are described by the differential relation

A.1

so that

A.2

At near-criticality, *f̅* splits into background and singular contributions:

A.3

The above definitions allow to express the former to leading order as

A.4

where we have used that *m*_*c*_ ≡ 0 while asymptotically close to criticality . On the other hand,  obeys *scaling*:^47^

A.5

where *Q*_*I*_ > 0 is a nonuniversal (i.e., system-dependent) critical amplitude and *W*_±_(*w*) is the universal scaling function at (+) *t̃* > 0 and (−) *t̃* < 0 depending on the scaling variable

A.6

with *U*_*I*_ > 0 another nonuniversal amplitude.

A small-*w* expansion of the scaling function applies to the critical isochore for *T* > *T*_*c*_:^48^

A.7

which leads to

A.8

with γ satisfying the scaling relation Δ = β + γ.

With this preamble, we are ready to work out CC and SLG models. The CC one is known to have the following mapping into the Ising model:^22^

A.9

A.10

where *t* ≡ (*T* – *T*_*c*_)/*T*_*c*_, , and , while , with *ℏ* the reduced Planck constant, is the thermal de Broglie wavelength for a particle with mass *m*. Then, introduction of eqs A.3 to A.5 into eq A.9 and eq A.10 leads to

A.11

where  has been obtained with the aid of Gibbs–Duhem relation

A.12

In turn, eq A.12 allows us to write

A.13

which combined with eq A.2 and eq A.11 straightforwardly leads to

A.14

It comes out from eq A.14 that the critical isochore, *v* = *v*_*c*_, is defined by *m* = 0 (and so *h* = 0). Thus, in the one-phase region, *T* > *T*_*c*_, eq A.8 applies and evaluation of the corresponding derivatives of *m* in eq A.14 leads to , , and  as defined by eq 3. This requires the use of exact differential relations involving χ_*hh*_ as given by eq A.8 and of derivatives of *h* with respect to *T* and *p* as readily obtained from eq A.10. The final result for the amplitudes is that quoted by eq 6 in subsection 3.4.

As for the SLG, we start from^41^

A.15

A.16

A procedure completely analogous to the one for the CC model above yields

A.17

A.18

A.19

with . This leads to eq 8 in subsection 3.4 for , , and .

## Supporting Information Available

Molar volumes and enthalpies (PDF)

## References

[ref1] Poole P. H.; Sciortino F.; Essmann U.; Stanley H. E. Phase Behavior of Metastable Water. Nature (London) 1992, 360, 324–328. 10.1038/360324a0.

[ref2] Speedy R. J.; Angell C. A. Isothermal Compressibility of Supercooled Water and Evidence for a Thermodynamic Singularity at −45 Degrees C. J. Chem. Phys. 1976, 65, 851 10.1063/1.433153.

[ref3] Angell C. A.; Oguni M.; Sichina W. J. Heat Capacity of Water at Extremes of Supercooling and Superheating. J. Phys. Chem. 1982, 86, 998 10.1021/j100395a032.

[ref4] Hare D. E.; Sorensen C. M. The Density of Supercooled Water. 2. Bulk Samples Cooled to the Homogeneous Nucleation Limit. J. Chem. Phys. 1987, 87, 4840 10.1063/1.453710.

[ref5] Holten V.; Qiu C.; Guillerm E.; Wilke M.; Ric̆ka J.; Frenz M.; Caupin F. Compressibility Anomalies in Stretched Water and their Interplay with Density Anomalies. J. Phys. Chem. Lett. 2017, 8, 5519–5522. 10.1021/acs.jpclett.7b02563. 29043801

[ref6] Kim K. H.; Spah A.; Pathak H.; Perakis F.; Mariedahl D.; Amann-Winkel K.; Sellberg J. A.; Lee J. H.; Kim S.; Park J.; et al. A. Maxima in the Thermodynamic Response and Correlation Functions of Deeply Supercooled Water. Science 2017, 358, 1589–1593. 10.1126/science.aap8269. 29269472

[ref7] Pathak A.; Spah A.; Esmaeildoost N.; Sellberg J. A.; Kim K. H.; Perakis F.; Amann-Winkel K.; Ladd-Parada M.; Koliyadu J.; Lane T. J.; et al. Enhancement and Maximum in the Isobaric Specific-Heat Capacity Measurements of Deeply Supercooled Water using Ultrafast Calorimetry. Proc. Natl. Acad. Sci. U. S. A. 2021, 118, e2018379118 10.1073/pnas.2018379118. 33526683 PMC8017957

[ref8] Palmer J. C.; Poole P. H.; Sciortino F.; Debenedetti P. G. Advances in Computational Studies of the Liquid-Liquid Transition in Water and Water-Like Models. Chem. Rev. 2018, 118, 9129–9151. 10.1021/acs.chemrev.8b00228. 30152693

[ref9] Liu Y.; Panagiotopoulos A. Z.; Debenedetti P. G. Low-Temperature Fluid-Phase Behavior of ST2 Water. J. Chem. Phys. 2009, 131, 104508 10.1063/1.3229892.

[ref10] Liu Y.; Palmer J. C.; Panagiotopoulos A. Z.; Debenedetti P. G. Liquid-Liquid Transition in ST2 Water. J. Chem. Phys. 2012, 137, 214505 10.1063/1.4769126. 23231249

[ref11] Palmer J. C.; Martelli F.; Liu Y.; Car R.; Panagiotopoulos A. Z.; Debenedetti P. G. Metastable Liquid-Liquid Transition in a Molecular Model of Water. Nature (London) 2014, 510, 385–388. 10.1038/nature13405. 24943954

[ref12] Ni Y.; Skinner J. L. Evidence for a Liquid-Liquid Critical Point in Supercooled Water within the E3B3Model and a Possible Interpretation of the Kink in the Homogeneous Nucleation Line. J. Chem. Phys. 2016, 144, 214501 10.1063/1.4952991. 27276957

[ref13] Hestand N. J.; Skinner J. L. Crossing the Widom Line in No-Man’s Land: Experiments, Simulations, and the Location of the Liquid-Liquid Critical Point in Supercooled Water. J. Chem. Phys. 2018, 149, 140901 10.1063/1.5046687. 30316289

[ref14] Debenedetti P. G.; Sciortino F.; Zerze G. Second Critical Point in Two Realistic Models of Water. Science 2020, 369, 289–292. 10.1126/science.abb9796. 32675369

[ref15] Weis J.; Sciortino F.; Panagiotopoulos A. Z.; Debenedetti P. G. Liquid-Liquid Criticality in the WAIL Water Model. J. Chem. Phys. 2022, 157, 024502 10.1063/5.0099520. 35840388

[ref16] Gartner T. E.; Piaggi P. M.; Car R.; Panagiotopoulos A. Z.; Debenedetti P. G. Liquid-Liquid Transition in Water from First Principles. Phys. Rev. Lett. 2022, 129, 255702 10.1103/PhysRevLett.129.255702. 36608224

[ref17] Gallo P.; Sciortino F. Ising Universality Class for the Liquid-Liquid Critical Point of a One-Component Fluid: A Finite-Size Scaling Study. Phys. Rev. Lett. 2012, 109, 177801 10.1103/PhysRevLett.109.177801. 23215223

[ref18] Griffiths R. B.; Wheeler J. C. Critical Points in Multicomponent Systems. Phys. Rev. A 1970, 2, 1047–1064. 10.1103/PhysRevA.2.1047.

[ref19] Cerdeiriña C. A.; Troncoso J.; González-Salgado D.; Debenedetti P. G.; Stanley H. E. Water’s Two-Critical-Point Scenario in the Ising Paradigm. J. Chem. Phys. 2019, 150, 244509 10.1063/1.5096890. 31255058

[ref20] Lemmon E. W.; Bell I. H.; Huber M. L.; McLinden M. O. Thermophysical Properties of Fluid Systems. In NIST Standard Reference Database Number 69; Linstrom P. J., Mallard W. G., Eds.; National Institute of Standards and Technology: Gaithesburg, MD, 20899; obtained on 15 January 2023 from http://webbook.nist.gov/chemistry.

[ref21] Lee T. D.; Yang C. N. Statistical Theory of Equations of State and Phase Transitions. 2. Lattice Gas and Ising Model. Phys. Rev. 1952, 87, 410–419. 10.1103/PhysRev.87.410.

[ref22] Cerdeiriña C. A.; Stanley H. E. Ising-like Models with Energy-Volume Coupling. Phys. Rev. Lett. 2018, 120, 120603 10.1103/PhysRevLett.120.120603. 29694060

[ref23] Holten V.; Bertrand C. E.; Anisimov M. A.; Sengers J. V. Thermodynamics of Supercooled Water. J. Chem. Phys. 2012, 136, 094507 10.1063/1.3690497. 22401452

[ref24] Luo J.; Xu L.; Lascaris E.; Stanley H. E.; Buldyrev S. V. Behavior of the Widom Line in Critical Phenomena. Phys. Rev. Lett. 2014, 112, 135701 10.1103/PhysRevLett.112.135701. 24745439

[ref25] Abascal J. L. F.; Vega C. A General Purpose Model for the Condensed Phases of Water: TIP4P/2005. J. Chem. Phys. 2005, 123, 234505 10.1063/1.2121687. 16392929

[ref26] Allen M. P.; Tildesley D. J. Computer Simulation of Liquids; Clarendon Press: Oxford, 1987.

[ref27] Frenkel D.; Smit B. Understanding Molecular Simulation; Elsevier: Orlando, 2002.

[ref28] van der Spoel D.; Lindahl E.; Hess B.; Groenhof G.; Mark A. E.; Berendsen H. J. C. GROMACS: Fast, Flexible, and Free. J. Comput. Chem. 2005, 26, 1701–1718. 10.1002/jcc.20291. 16211538

[ref29] Parrinello M.; Rahman A. Polymorphic Transitions in Single Crystals: A New Molecular Dynamics Method. J. Appl. Phys. 1981, 52, 7182–7190. 10.1063/1.328693.

[ref30] Nosé S. A Molecular Dynamics Method for Simulations in the Canonical Ensemble. Mol. Phys. 1984, 52, 255–268. 10.1080/00268978400101201.

[ref31] Hoover W. G. Canonical Dynamics: Equilibrium Phase-space Distributions. Phys. Rev. A 1985, 31, 1695–1697. 10.1103/PhysRevA.31.1695. 9895674

[ref32] Essmann U.; Perera L.; Berkowitz M. L.; Darden T.; Lee H.; Pedersen L. G. A Smooth Particle Mesh Ewald Method. J. Chem. Phys. 1995, 103, 8577–8593. 10.1063/1.470117.

[ref33] Blume M. Theory of First-Order Magnetic Phase Change in UO_2_. Phys. Rev. 1966, 141, 517–524. 10.1103/PhysRev.141.517.

[ref34] Capel H. W. On the Possibility of First-Order Transitions in Ising Systems of Triplet Ions with Zero-Field Splitting. Physica 1966, 32, 966–988. 10.1016/0031-8914(66)90027-9.

[ref35] Blume M.; Emery V. J.; Grifiths R. B. Ising Model for the Lambda Transition and Phase Separation in He^3^-He^4^ Mixtures. Phys. Rev. A 1971, 4, 1071–1077. 10.1103/PhysRevA.4.1071.

[ref36] Ciach A.; Gozdz W.; Perera A. Simple Three-State Lattice Model for Liquid Water. Phys. Rev. E 2008, 78, 011203 10.1103/PhysRevE.78.021203. 18850823

[ref37] Caupin F.; Anisimov M. A. Minimal Microscopic Model for Liquid Polyamorphism and Waterlike Anomalies. Phys. Rev. Lett. 2021, 127, 185701 10.1103/PhysRevLett.127.185701. 34767396

[ref38] Abascal J. L. F.; Vega C. Widom Line and the Liquid-Liquid Critical Point for the TIP4P/2005 Water Model. J. Chem. Phys. 2010, 133, 234502 10.1063/1.3506860. 21186870

[ref39] Kim Y. C.; Fisher M. E.; Orkoulas G. Asymmetric Fluid Criticality. I. Scaling with Pressure Mixing. Phys. Rev. E 2003, 67, 061506 10.1103/PhysRevE.67.061506. 16241232

[ref40] Abdulagatov A. I.; Stepanov G. V.; Abdulagatov I. M. Vapor-Pressure for the Pure Fluids from Calorimetric Measurements Near the Critical Point. Fluid Phase Equilib. 2003, 209, 55–79. 10.1016/S0378-3812(03)00083-9.

[ref41] Cerdeiriña C. A.; Orkoulas G. Compressible Cell Gas Models for Asymmetric Fluid Criticality. Phys. Rev. E 2017, 95, 032105 10.1103/PhysRevE.95.032105. 28415250

[ref42] Luo J.; Xu L.; Angell C. A.; Stanley H. E.; Buldyrev S. V. Physics of the Jagla Model as the Liquid-Liquid Coexistence Line Slope Varies. J. Chem. Phys. 2015, 142, 224501 10.1063/1.4921559. 26071714

[ref43] Holten V.; Palmer J. C.; Poole P. H.; Debenedetti P. G.; Anisimov M. A. Two-State Thrmodynamics of the ST2Model of Supercooled Water. J. Chem. Phys. 2014, 140, 104502 10.1063/1.4867287. 24628177

[ref44] Jedrecy A.; Saitta A. M.; Pietrucci F. Free Energy Calculations and Unbiased Molecular Dynamics Targeting the Liquid-Liquid Transition in Water No Man’s Land. J. Chem. Phys. 2023, 158, 014502 10.1063/5.0120789. 36610960

[ref45] Kim K. H.; Amann-Winkel K.; Giovambattista N.; Spah A.; Perakis F.; Pathak H.; Parada M. L.; Yang C.; Mariedahl D.; Eklund T.; et al. Experimental Observation of the Liquid-Liquid Transition in Bulk Supercooled Water Under Pressure. Science 2020, 370, 978–982. 10.1126/science.abb9385. 33214280

[ref46] Amann-Winkel K.; Kim K. H.; Giovambattista N.; Parada M. L.; Spah A.; Perakis F.; Pathak H.; Yang C.; Mariedahl D.; Eklund T.; et al. Liquid-Liquid Phase Separation in Supercooled Water from Ultrafast Heating of Low-Density Amorphous Ice. Nat. Commun. 2023, 14, 442 10.1038/s41467-023-36091-1. 36707522 PMC9883474

[ref47] Widom B. Equation of State in the Neighborhood of the Critical Point. J. Chem. Phys. 1965, 43, 3898–3905. 10.1063/1.1696618.

[ref48] Fisher M. E.; Kim Y. C. Right and Wrong near Critical Endpoints. J. Chem. Phys. 2002, 117, 779–787. 10.1063/1.1481381.

