# Supplementary material for: Liquid–Liquid Criticality in TIP4P/2005 and Three-State Models of Water
Source: J Phys Chem B. 2023 Apr 25;127(17):3902–10. doi: 10.1021/acs.jpcb.3c00696 (PMC10165646; doi:10.1021/acs.jpcb.3c00696)
Supplement: Supplementary file 1 — jp3c00696_si_001.pdf [file jp3c00696_si_001.pdf]

SUPPORTING INFORMATION OF MANUSCRIPT

Liquid-Liquid Criticality in TIP4P/2005 and Three-State Models of Water

Claudio A. Cerdeiriña,* Diego González-Salgado, and Jacobo Troncoso

Departamento de Física Aplicada and Instituto de Física y Ciencias Aeroespaciales, Universidad de Vigo—Campus del Agua, Ourense 32004, Spain

*Corresponding author (calvarez@uvigo.es)

Tables S1 to S10 contain density and enthalpy data for TIP4P/2005 water at the simulated temperatures and pressures.

Table S.1. Molar volume *v* of TIP4P/2005 water at temperature *T* and pressure *p*.

	p (bar)					
	350	400	430	440	450	500
T (K)	*v* ($cm^3 \cdot mol^{-1}$)					
240						17.7310
245						17.6848
250				17.7032	17.6935	17.6484
255			17.6855	17.6753	17.6677	17.6231
260			17.6668	17.6574	17.6498	
265	17.7243	17.6819	17.6564	17.6483	17.6396	17.5988
270	17.7193	17.6779	17.6536	17.6450	17.6376	17.5972
275	17.7207	17.6801	17.6566	17.6488	17.6403	17.6015
280			17.6647	17.6571	17.6490	17.6112
285				17.6706	17.6628	17.6250
290					17.6810	17.6439
295						17.6663

Table S.2. Molar enthalpy *h* of TIP4P/2005 water at temperature *T* and pressure *p*.

	p (bar)					
	350	400	430	440	450	500
T (K)	*h* ($J \cdot mol^{-1}$)					
240						–44874.6
245						–44400.1
250				–44042.6	–44023.4	–43927.1
255			–43594.0	–43574.2	–43556.2	–43462.9
260			–43131.8	–43112.3	–43094.6	
265	–42817.7	–42726.9	–42673.0	–42654.7	–42636.7	–42546,9
270	–42360.8	–42271.4	–42219.1	–42201.1	–42183.8	–42095,6
275	–41907.7	–41820.1	–41767.5	–41750.9	–41733.2	–41647,6
280			–41320.1	–41303.7	–41285.9	–41201.6
285				–40859.2	–40842.3	–40758.9
290					–40401.2	–40318.7
295						–39881.2

Table S.3. Molar volume *v* of TIP4P/2005 water at temperature *T* and pressure *p*.

	p (bar)					
	530	540	550	600	630	640
T (K)	*v* ($cm^3 \cdot mol^{-1}$)					
225				17.8635		
230			17.8241	17.7635		
235			17.7409	17.6847		
240			17.6781	17.6277		
245			17.6336	17.5857		
250		17.6100	17.6019	17.5561		17.5199
255	17.5960	17.5878	17.5785	17.5361	17.5098	17.5005
260	17.5814	17.5739	17.5646		17.4991	17.4908
265	17.5741	17.5664	17.5580	17.5178	17.4937	17.4864
270	17.5736	17.5662	17.5578	17.5189	17.4957	17.4877
275	17.5785	17.5713	17.5631	17.5253	17.5030	17.4954
280	17.5888	17.5809	17.5734	17.5363	17.5141	17.5066
285		17.5954	17.5881	17.5513		17.5226
290			17.6069	17.5705		
295				17.5938		

Table S.4. Molar enthalpy *h* of TIP4P/2005 water at temperature *T* and pressure *p*.

	p (bar)					
	530	540	550	600	630	640
T (K)	*h* ($J \cdot mol^{-1}$)					
225				–46147.2		
230			–45753.6	–45643.3		
235			–45256.2	–45150.4		
240			–44772.4	–44673.1		
245			–44298.5	–44201.5		
250		–43851.1	–43832.0	–43737.7		–43661.9
255	–43406.9	–43388.4	–43370.5	–43278.4	–43223.6	–43204.5
260	–42949.0	–42930.1	–42912.3		–42768.4	–42751.7
265	–42494.2	–42476.6	–42458.5	–42370.7	–42317.6	–42300.5
270	–42043.4	–42025.8	–42007.8	–41921.1	–41869.3	–41852.2
275	–41595.8	–41578.6	–41560.7	–41475.9	–41424.7	–41408.2
280	–41150.7	–41134.4	–41117.3	–41033.1	–40982.8	–40966.1
285		–40691.7	–40675.6	–40592.7		–40526.7
290			–40236.3	–40154.7		
295				–39718.9		

Table S.5. Molar volume *v* of TIP4P/2005 water at temperature *T* and pressure *p*.

	p (bar)					
	650	700	750	800	850	900
T (K)	*v* ($cm^3 \cdot mol^{-1}$)					
190		18.6845		18.6023		18.5199
195		18.6625		18.5906		18.4758
200		18.5993		18.5146		18.3462
205		18.4381		18.2804		18.0725
210		18.2374		18.0470		17.8667
215		18.0241		17.8453		17.6878
220	17.9300	17.8524	17.7803	17.7066	17.6386	17.5737
225	17.7972	17.7353	17.6649	17.6045	17.5453	17.4835
230	17.7027	17.6434	17.5857	17.5282	17.4730	17.4191
235	17.6301	17.5773	17.5253	17.4720	17.4201	17.3727
240	17.5766	17.5286	17.4774	17.4299		17.3385
245	17.5385	17.4923	17.4461			
250	17.5117	17.4664				
255	17.4925	17.4512				
260	17.4825					
265	17.4791					
270	17.4810					
275	17.4876					
280	17.4995	17.4635				
285	17.5156	17.4794				
290	17.5352	17.5000				
295		17.5235				

Table S.6. Molar enthalpy *h* of TIP4P/2005 water at temperature *T* and pressure *p*.

	p (bar)					
	650	700	750	800	850	900
T (K)	*h* (J·mol^{-1})					
190		–49410.1		–49210.7		–49004.8
195		–49065.2		–48879.7		–48621.9
200		–48663.9		–48441.5		–48150.9
205		–48136.8		–47854.5		–47554.8
210		–47564.1		–47266.7		–46997.4
215		–46977.1		–46707.7		–46463.6
220	–46553.5	–46429.7	–46309.9	–46191.2	–46078.2	–45970.8
225	–46031.5	–45920.5	–45804.4	–45697.9	–45593.5	–45485.8
230	–45533.1	–45426.9	–45321.4	–45217.2	–45115.0	–45014.9
235	–45048.8	–44947.0	–44845.6	–44745.9	–44646.7	–44552,3
240	–44573.4	–44475.4	–44377.3	–44280.9		–44094,0
245	–44106.7	–44010.8	–43916.2			
250	–43644.9	–43550.4				
255	–43186.7	–43095.6				
260	–42733.1					
265	–42282.1					
270	–41835.5					
275	–41391.0					
280	–40949.2	–40865.7				
285	–40509.9	–40427.2				
290	–40073.0	–39992.2				
295		–39558.6				

Table S.7. Molar volume *v* of TIP4P/2005 water at temperature *T* and pressure *p*.

	p (bar)					
	1000	1100	1200	1300	1400	1500
T (K)	*v* ($cm^3 \cdot mol^{-1}$)					
190	18.4305	18.3481	18.1773	17.9606	17.6963	17.3430
195	18.3559	18.2628	17.9819	17.7252	17.4365	17.1764
200	18.1503	17.9207	17.6951	17.4799	17.2546	17.0899
205	17.9015	17.6742	17.4801	17.3040	17.1532	17.0014
210	17.6925	17.5199	17.3558	17.2099	17.0762	16.9561
215	17.5359	17.3960	17.2634	17.1400	17.0231	16.9179
220	17.4380	17.3150	17.1974	17.0881	16.9837	16.8893

Table S.8. Molar enthalpy *h* of TIP4P/2005 water at temperature *T* and pressure *p*.

	p (bar)					
	1000	1100	1200	1300	1400	1500
T (K)	*h* ($J \cdot mol^{-1}$)					
190	–48812.2	–48598.2	–48346.0	–48076.5	–47776.9	–47471.3
195	–48394.6	–48190.4	–47839.7	–47553.5	–47260.7	–47006.2
200	–47859.5	–47564.4	–47279.6	–47018.5	–46772.6	–46558.0
205	–47289.7	–47006.7	–46756.3	–46522.3	–46311.2	–46107.7
210	–46747.8	–46503.6	–46272.6	–46060.9	–45858.3	–45664.3
215	–46232.5	–46012.6	–45805.9	–45603.3	–45408.7	–45225.0
220	–45752.9	–45543.7	–45344.5	–45148.8	–44964.2	–44784.3

Table S.9. Molar volume *v* of TIP4P/2005 water at temperature *T* and pressure *p*.

	p (bar)		
	1772	1872	1972
T (K)	*v* ($cm^3·mol^{-1}$)		
185	16.8347	16.6559	16.5252
190	16.7889	16.6388	16.5213
195	16.7379	16.6189	16.5122
200	16.7098	16.6034	16.5109
205	16.6887	16.5977	16.5074
210	16.6752	16.5869	16.5027
215	16.6648	16.5806	16.5048
220	16.6565	16.5823	16.5065
225		16.5811	
230	16.6533	16.5838	16.5178
235		16.5889	
240	16.6619	16.5970	16.5351
245			
250	16.6798	16.6168	16.5611
255			
260	16.7079	16.6477	16.5945
265			
270	16.7454	16.6870	16.6362
275			
280	16.7902	16.7333	16.6836
285			
290	16.8431	16.7873	16.7381
295			
300	16.9030	16.8478	16.7992

Table S.10. Molar enthalpy h of TIP4P/2005 water at temperature T and pressure p.

	p (bar)		
	1772	1872	1972
T (K)		h (J·mol^{-1})	
185	–47302.3	–47116.3	–46946.3
190	–46885.9	–46706.4	–46532.6
195	–46460.6	–46284.6	–46117.5
200	–46034.9	–45860.6	–45690.1
205	–45606.7	–45434.7	–45267.3
210	–45174.6	–45004.5	–44839.4
215	–44743.4	–44573.9	–44409.8
220	–44312.2	–44145.4	–43978.8
225		–43712.4	
230	–43444.5	–43280.4	–43117.3
235		–42846.9	
240	–42577.6	–42415.2	–42253.9
245			
250	–41713.8	–41553.4	–41395.1
255			
260	–40854.4	–40696.9	–40539.9
265			
270	–40000.6	–39844.8	–39689.9
275			
280	–39152.6	–38999.0	–38845.6
285			
290	–38311.1	–38160.0	–38009.2
295			
300	–37477.6	–37327.7	–37178.5
